# Prognostic impact of neutrophil-to-lymphocyte ratio in gliomas: a systematic review and meta-analysis

**DOI:** 10.1186/s12957-019-1686-5

**Published:** 2019-08-31

**Authors:** Yu-ying Lei, Yi-tong Li, Qi-lu Hu, Juan Wang, Ai-xia Sui

**Affiliations:** Department of Oncology, Hebei General Hospital, Shijiazhuang, 050051 Hebei China

**Keywords:** Neutrophil-to-lymphocyte ratio, Gliomas, Prognosis, Meta-analysis

## Abstract

**Background:**

In some malignant tumors, a high neutrophil-to-lymphocyte ratio (NLR) is connected with unfavorable prognosis. Nevertheless, the prognostic value of the NLR in gliomas remains disputed. The clinical significance of the NLR in gliomas was investigated in our study.

**Methods:**

The databases, PubMed, Embase, and the Cochrane Library, were searched using words like “glioma,” “glioblastoma,” “neutrophil-to-lymphocyte ratio,” and others through May 2019. We evaluated the significance of NLR on overall survival (OS) of patients with gliomas in our study.

**Results:**

Finally, 16 cohorts with 2275 patients were analyzed. The pooled analysis revealed that an elevated NLR was connected with unfavorable OS (hazards ratio (HR): 1.43, 95% confidence interval (CI): 1.27–1.62) outcomes of patients with gliomas.

**Conclusion:**

A high NLR can be considered a high-risk prognostic factor in gliomas, and more adjuvant chemotherapy should be recommended for high-risk patients.

**Electronic supplementary material:**

The online version of this article (10.1186/s12957-019-1686-5) contains supplementary material, which is available to authorized users.

## Background

Gliomas are the most frequent type of cerebral tumors. Approximately, 81% of primary intracranial tumors are gliomas [1]. The most challenging malignant glioma is glioblastoma (GBM; WHO grade IV); patients with GBM only have a median survival time of 14.6 months [1]. Despite the improvements in the multimodality treatment (maximal safe resection, radiation therapy concurrent with temozolomide, and subsequent adjuvant temozolomide chemotherapy) [2, 3], local recurrence and metastasis remain significant concerns in most patients. Therefore, it is necessary to identify biological markers for estimating the progression or survival of patients with glioma.

In clinical practice, traditional prognostic factors, including the Karnofsky performance status, tumor location, age at presentation, isocitrate dehydrogenase (IDH) status, and extent of surgery, have gradually proved to be insufficient and inaccurate. The identification of economically feasible and readily available prognostic biomarkers could assist us in identifying high-risk patients to determine the best treatment options and further improve the prognosis of the patients. Inflammatory factors have to be related to cancer initiation, progression, invasion, and metastasis [4, 5]. In several types of cancers, biomarkers of inflammatory reactions have been considered as prognostic factors [6]. As a type of inflammatory parameter, it is easy to obtain the peripheral blood neutrophil-to-lymphocyte ratio (NLR). Furthermore, in various cancers [7], an elevated NLR is considered as a poor prognostic factor. NLR is an important factor that influence prognosis in ovarian, colorectal, breast, pancreatic, urothelial, renal cell cancers, and myeloma patients [8–14]. Recently, elevated NLR was reported to be correlated with poor prognosis in patients with gliomas in several studies. However, the outcomes of published articles were inconsistent. Therefore, our study aimed to elucidate the clinical significance of NLR for gliomas.

## Methods

### Search strategy

The electronic databases, PubMed, Cochrane Library, and Embase, were searched from the time of their conception until May 2019. The databases were searched using the following words: (‘glioblastoma’ OR ‘glioma’) AND (‘neutrophil to lymphocyte ratio’ OR ‘neutrophil lymphocyte ratio’ OR ‘neutrophil-to-lymphocyte ratio’ OR ‘NLR’) AND (‘survival’ OR ‘mortality’ OR ‘outcome’ OR ‘prognostic’ OR ‘prognosis’). We manually screened the references of the related articles to expand the search range.

### Selection criteria

The inclusion criteria in our meta-analysis were as follows: (1) patients pathologically confirmed with gliomas, (2) the prognostic significance of peripheral blood NLR for gliomas was assessed, (3) cutoff of NLR was provided, and (4) hazard ratios (HRs) and 95% confidence intervals (CIs) for NLR on overall survival (OS) were available. The following studies were excluded: (1) case reports, letters, conference abstracts, non-clinical studies, and reviews without available data; (2) studies with insufficient information to evaluate HRs and 95% CIs; and (3) duplicated publications.

### Data extraction and quality assessment

Two investigators independently selected the studies that fulfilled our inclusion criteria and extracted the relevant information. The related information was extracted as follows: first author’s surname, country, sample size, age of the study population, publication year, histology, duration, treatment, cutoff value of NLR, sampling time, and HR and 95% CI for OS. Any disagreement was resolved through discussion.

Two reviewers used Newcastle–Ottawa quality assessment scale (NOS) [15] to evaluate the quality of studies. Using the NOS, the studies are evaluated on three ways, namely comparability, selection, and outcome confirmation. Each parameter also has subitems. The maximum score is nine stars, and NOS scores ≥ 5 is considered of high quality [15].

### Statistical analysis

The collected data from the included studies were combined using Review Manager 5.3 (The Cochrane Collaboration, Copenhagen, Denmark). Forest plots were constructed to assess the predictive role of NLR in gliomas. HRs and 95% CIs for OS were synthesized with a random effect model. A random effect model or fixed effect model was employed depending on the heterogeneity of the studies [16]. The heterogeneity was evaluated with the *I*^2^ statistic. The data were synthesized using a fixed effect model with *I*^2^ < 25%. In case of *I*^2^ > 25%, a random effect model was used for data synthesis. The sources of heterogeneity were evaluated by subgroup analysis. Sensitivity analysis was used to appraise the stability of the outcome. Funnel plots were constructed to evaluate publication bias. Statistical difference was defined as *P* value < .05.

## Results

### Description of the trials

**Fig. 1:**
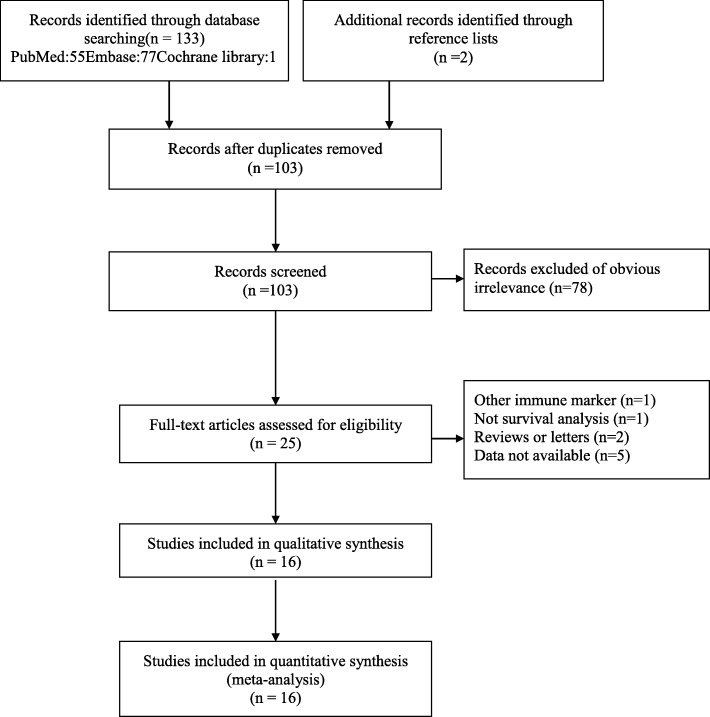
Procedure of study selection

A flow diagram based on the PRISMA statement (Additional file 1) summarizing the process of study retrieval is illustrated in Fig. 1. A total of 16 articles published between 2013 and 2019 were incorporated in our study [17–32]. The data of 2275 patients in whom the prognostic significance of NLR was assessed were included. The demographic data of the patients in the included trials is shown in Table 1. The NOS scoring details are presented in Additional file 2. There were 2 studies from USA, 8 from China, 1 from Canada, 1 from Russia, 2 from Turkey, 1 from Singapore, and 1 from Portugal. All trials were retrospective ones. The cutoff values ranged from 2.5 to 7.5 in the included trials, with an average value of 4.03. Eleven studies used NLR from the preoperative blood sample, whereas 2 used NLR from the postoperative blood sample. Thirteen of the 15 trials applied multivariate analysis. The NOS scores ranged from 5 to 7. The average number of NOS scores was 5.375.

**Table 1:** Baseline Characteristics of Included Studies

Study	Year	Country	Duration	Histology	Sample size (Femal/Male)	Age	Treatment	Sampling time	NLR Cut-off value	Outcome	NOS score
Bambury et al. [17]	2013	USA	2004-2013	glioblastoma multiforme	84 (19/65)	median 58	S+R+C	pretreatment	4	multivariate	7
Han et al. [18]	2015	China	2010-2014	glioblastoma	152 (57/95)	mean 50	NR	pretreatment	4	multivariate	6
Auezova et al. [19]	2016	Russia	2009-2012	gliomas	178 (85/93)	mean 41	NR	pretreatment	4	univariate	5
S.S.Q. et al. [29]	2017	Singapore	NR	glioblastoma	58 (NR)	NR	NR	perioperative	2.5	multivariate	5
Kaya et al. [20]	2017	Turkey	2011-2015	glioblastoma	90 (NR)	mean 55	S+R+C	pretreatment	5	multivariate	5
Lopes et al. [21]	2017	Portugal	2005-2013	glioblastoma multiforme	140 (42/98)	mean 62	S+C	pretreatment	5	multivariate	5
Matthew et al. [22]	2017	Canada	2004-2010	glioblastoma	369 (131/238)	median 55	S+R+C	postoperative	7.5	multivariate	5
Wang et al. [23]	2017	China	2009-2014	glioblastoma	166 (70/96)	mean 52	NR	pretreatment	4	multivariate	5
Wiencke et al. [24]	2017	USA	NR	glioma (grade II-IV)	72 (20/52)	median 47	NR	postoperative	4	multivariate	5
Bao et al. [28]	2018	China	2012-2017	glioma	219 (95/124)	NR	NR	pretreatment	2.5	multivariate	6
Wang et al. [25]	2018	China	2010-2013	glioma (grade I-IV)	112 (42/70)	mean 50	NR	pretreatment	4	multivariate	6
Weng et al. [26]	2018	China	2011-2014	glioblastoma	105 (52/53)	NR	S+R+C	pretreatment	4	multivariate	5
Yersal et al. [27]	2018	Turkey	2012-2017	glioblastoma	80 (41/39)	mean 56	S+R+C	pretreatment	4	univariate	5
Hao et al. [31]	2019	China	2012-2017	glioblastoma multiforme	187 (71/116)	mean 55	NR	NR	4.1	univariate	6
Yang et al. [30]	2019	China	2008-2012	glioblastoma multiforme	128 (57/71)	mean 55	S+R+C	NR	2.8	multivariate	5
Gan et al. [32]	2019	China	2014-2018	glioma (grade III-IV )	135 (46/89)	Mean70	S+R+C	pretreatment	3	multivariate	5

*NR* Not reported, *S* Surgery, *R* Radioation, *C* Chemotherapy, *NOS* Newcastle Ottawa Scale

### NLR and OS in patients with gliomas

**Fig. 2:**
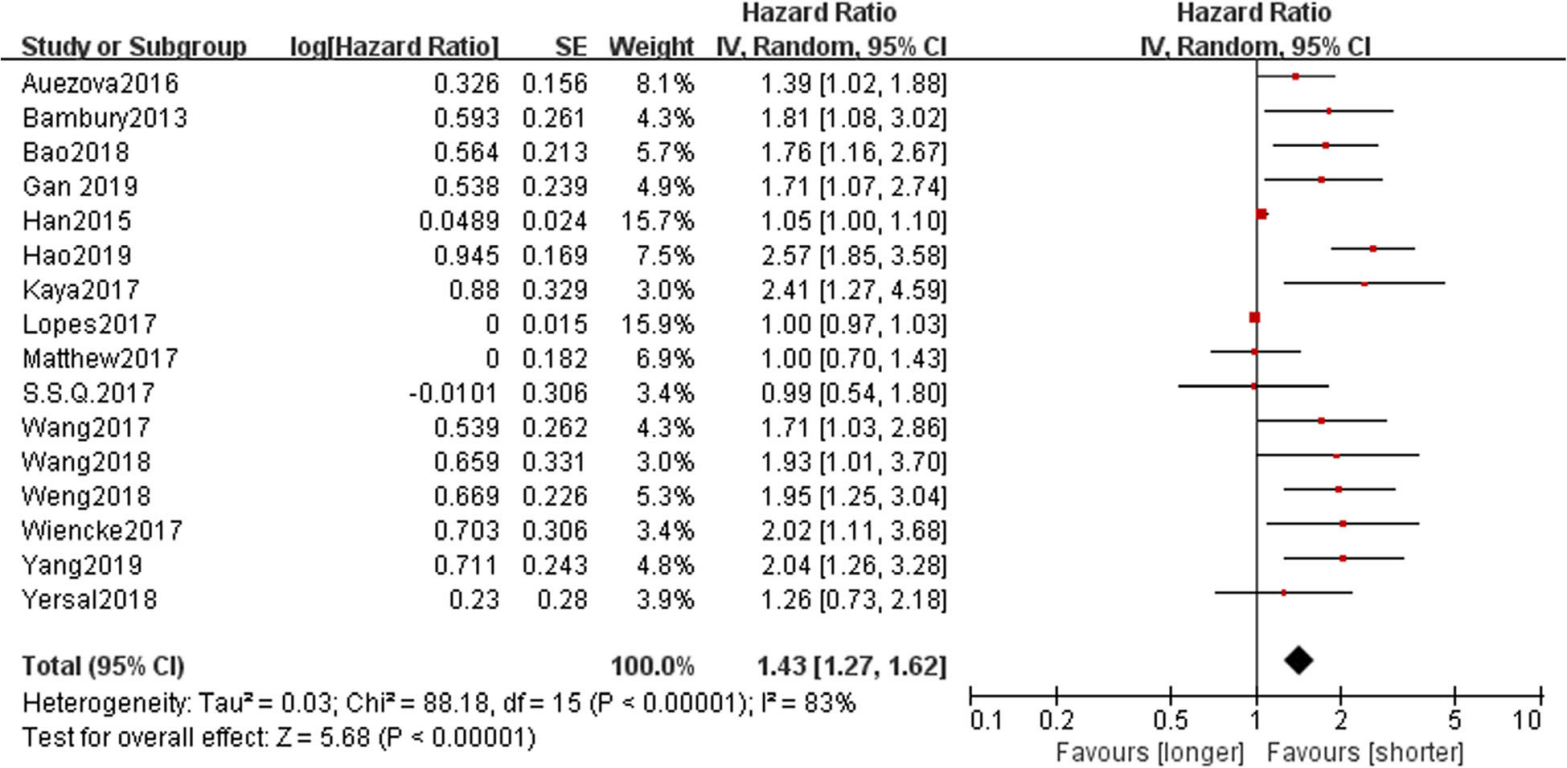
Hazard ratios (HRs) and 95% confidence intervals (CIs) in studies assessing the relationship between NLR and overall survival (OS) in patients with gliomas

A high preoperative NLR was connected with unfavorable OS (HR: 1.43, 95% CI: 1.27–1.62, *P* < 0.00001; Fig. 2) in patients with gliomas. The heterogeneity analysis among the studies showed an *I*^2^ value of 83% (*P* < 0.00001), which indicated obvious heterogeneity. A subgroup analysis was conducted on the basis of the latent confounding factors, such as histology, cutoff value of NLR, analysis method, ethnicity, NOS score, and sampling time. On stratification by ethnicity in the subgroup analysis, a low NLR predicted a positive prognosis in the Asian (HR: 1.64, 95% CI: 1.28–2.10), but not in the Caucasian (HR: 1.26, 95% CI: 0.92–1.72). Stratification by histology revealed that a low NLR predicted longer OS in trials with patients with gliomas of various grades (HR: 1.68, 95% CI: 1.40–2.01) and in those patients with GBM (HR: 1.29, 95% CI: 1.13–1.47). Furthermore, the subgroup analysis according to the cutoff value of NLR indicated that a high NLR was connected with negative OS in patients with gliomas in trials with cutoff value of NLR = 4 (HR: 1.55, 95% CI: 1.22–1.97) and in those in trials with cutoff values of NLR ≠ 4 (HR: 1.52, 95% CI: 1.06–2.19). Results of the subgroup analysis on the basis of the NOS score suggested that high NLR was connected with poor OS when the NOS score was ≤ 5 (HR: 1.46, 95% CI: 1.17–1.83) and NOS score was > 5 (HR: 1.72, 95% CI: 1.09–2.72). Results of the subgroup analysis on the basis of the analysis method showed that a low NLR represented good prognostic significance in both the univariate analysis (HR: 1.69, 95% CI: 1.06–2.67) and multivariate analysis (HR: 1.31, 95% CI: 1.16–1.48). Finally, analysis on the subgroup of sampling time indicated that an elevated NLR was connected with negative OS in gliomas with preoperative blood sampling (HR: 1.29, 95% CI: 1.14–1.45), but not in those with postoperative blood sampling (HR: 1.36, 95% CI: 0.69–2.70) (Table 2).

**Table 2:** Subgroup analysis of the association between NLR and OS

Factors	No. of studies	No. of patients	HR (95% CI)	*P*	Heterogeneity	
					*I*^2^ (%)	*P*_h_
Overall	16	2275	1.43 (1.27–1.62)	< 0.00001	83	< 0.00001
Ethnicity						
Caucasian	4	665	1.26 (0.92–1.72)	0.16	71	0.02
Asian	12	1610	1.64 (1.28–2.10)	< 0.0001	83	< 0.00001
Histology						
Glioblastoma	10	1431	1.29 (1.13–1.47)	0.0002	84	< 0.00001
Glioma(various grades)	6	844	1.68 (1.40–2.01)	< 0.00001	0	0.74
Cutoff value						
= 4	9	1168	1.55 (1.22–1.97)	0.0003	74	0.0002
≠ 4	7	1107	1.52 (1.06–2.19)	0.02	88	< 0.00001
NOS score						
≤ 5	11	1521	1.46 (1.17–1.83)	0.001	77	< 0.00001
> 5	5	754	1.72 (1.09–2.72)	0.02	90	< 0.00001
Analysis method						
Univariate	3	445	1.69 (1.06–2.67)	0.03	77	0.01
Multivariate	13	1830	1.31 (1.16–1.48)	< 0.0001	78	< 0.00001
Sampling time						
Preoperative	11	1461	1.29 (1.14–1.45)	< 0.0001	78	< 0.00001
Postoperative	2	441	1.36 (0.69–2.70)	0.38	74	0.05

*HR* hazard ratio, *CI* confidence interval, *P P* value for statistical significance based on *Z* test, *P*_*h*_ *P* value for heterogeneity based on *Q* test

### Sensitivity analysis and publication bias

To appraise the impact of each research on the overall outcome (HR) of OS, a sensitivity analysis was conducted. As for the HR on overall survival, we removed each study individually and the HR value or degree of significance did not substantially change.

**Fig. 3:**
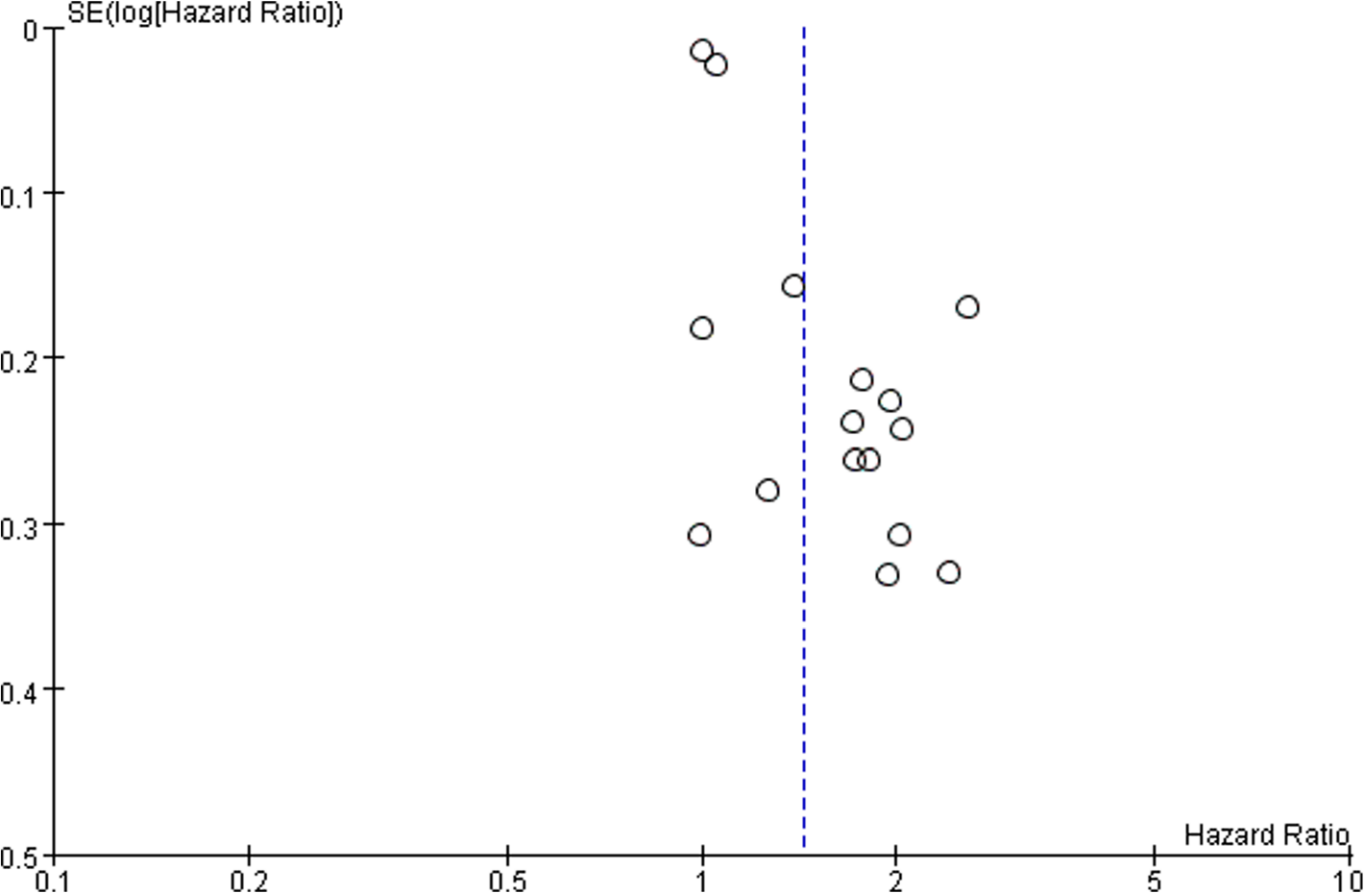
Funnel plot of publication bias test for OS in patients with gliomas

The shape of the funnel plots showed asymmetry and indicated significant publication bias in OS (Fig. 3).

## Discussion

To illuminate the relationship between NLR and gliomas, we conducted a meta-analysis by consolidating the published literature. In the current study, we incorporated 16 studies with 2275 patients to assess the clinical significance of NLR in gliomas. Our study indicated that a high preoperative NLR was connected with unfavorable OS in gliomas.

The role of NLR has been studied in other cancers, including colorectal, ovarian, breast, pancreatic, urothelial, and renal cell cancers, myeloma, and others [8–14]. The results of our pooled analysis are in agreement with results from these abovementioned studies on other cancers.

The mechanisms behind the relationship between a high NLR and unfavorable OS in gliomas have not been clearly illuminated. One possible mechanism could be the relationship between NLR and inflammatory response. A high NLR indicates relative neutrophilia and lymphopenia. Neutrophilia inhibits immune cells such as lymphocytes, natural killer cells, and activated T cells [33, 34]. This could stimulate the proliferation of cancer cells. On the other hand, in several studies, the role of lymphocytes has been presented, showing that lymphocyte infiltration of tumor cells has been associated with better response to treatment [35]. Thus, NLR might be regarded as a crude measure to reflect the balance between immunocytes and neutrophils. Moreover, it is an easily obtained and cost-effective index in clinical work, thus making it an attractive prognostic index for gliomas.

Notably, half of included articles in our study are from China; therefore, we performed a subgroup analysis of the studies based on ethnicity, to explore whether race had any effect on the outcome. On stratification by ethnicity in the subgroup analysis, a low NLR predicted a positive prognosis in the Asians, but not in the Caucasians. Twelve of the 16 included studies were from Asia, which may cause selection bias. In the future, relevant trials are needed to provide further evidence for the prognostic significance of NLR on race. In addition, analysis on the basis of sampling time indicated that an elevated NLR was connected with negative OS in gliomas with preoperative blood sampling, but not in those with postoperative blood sampling. Complex factors may influence the NLR value. It is suggested that pretreatment NLR should be used to estimate prognosis in clinical practice.

In our study, there are some limitations. First, all of incorporated trials are retrospective ones. Second, because of lack of individual patient data, the optimal NLR cutoff value could not be provided for clinical practice. Future studies are needed to explore the best NLR cutoff value. Third, the study has a publication bias. As mentioned previously, an obvious bias in the OS for glioma patients is present. There may be various factors contributing to publication bias. In my view, apart from the factors such as termination of publication and negative results not being published, the language limitation may be the main factor, as our searching language was mainly English. Finally, detailed information of unknown pretreatment (i.e., physical conditions, comorbidities, infective symptoms, medication, hypertension, lifestyle habits, and diabetes mellitus) could influence the NLR value, thus weakening its actual relationship with cancer-specific endpoints.

Despite these limitations, some advantages of our meta-analysis exist. First, most of the data were obtained from multivariate analysis, with three studies providing univariate outcomes. In our subgroup analysis, a low NLR represented good prognostic significance for glioma patients both with the univariate and multivariate analysis methods (Table 2). Additionally, NLR is an easily available biomarker. It can be obtained during the routine checkup. It is also an ideal index as obtaining it is economically cheap and fast. In the future, well-designed prospective trials with longer follow-up periods and further confirmatory trials are needed to provide further evidence of the prognostic significance of NLR in screening high-risk patients with gliomas.

## Conclusion

In conclusion, a high NLR is related to poor survival in patients with gliomas. NLR may serve as a cost-effective prognostic biomarker to identify high-risk patients who might need further therapy. More high-quality prospective trials are needed to assess the practicability of NLR in gliomas.

## Additional files

Additional file 1: PRISMA 2009 checklist. (DOC 66 kb)

Additional file 2: Newcastle-Ottawa scale (NOS). (DOC 46 kb)

## Data Availability

The datasets used and/or analyzed during the current study are available from the corresponding author on reasonable request.

## Abbreviations

CI: Confidence interval
GBM: Glioblastoma
HR: Hazard ratio
IDH: Isocitrate dehydrogenase
NLR: Neutrophil-to-lymphocyte ratio
NOS: Newcastle–Ottawa quality assessment scale
OS: Overall survival
