# Supplementary material for: Prognostic impact of neutrophil-to-lymphocyte ratio in gliomas: a systematic review and meta-analysis
Source: World J Surg Oncol. 2019 Aug 31;17:152. doi: 10.1186/s12957-019-1686-5 (PMC6717646; doi:10.1186/s12957-019-1686-5)
Supplement: Supplementary file 2 — Newcastle-Ottawa scale (NOS). (DOC 46 kb) [file 12957_2019_1686_MOESM2_ESM.doc]

**Additional file 2.** Newcastle-Ottawa Scale (NOS) Table: Methodological Quality Of Cohort Studies Included In The Meta-Analysis*

| **Study** | **Representativeness**  **of the exposed cohort** | **Selection of the unexposed**  **cohort** | **Ascertainment**  **of exposure** | **Outcome of interest**  **not present**  **at start of study** | **Control for**  **important factor or additional factor** | **Assessment of outcome** | **Follow-up**  **long enough for outcomes**  **to occur** | | **Adequacy of**  **follow-up**  **of cohorts** | **Total quality**  **scores** |
| --- | --- | --- | --- | --- | --- | --- | --- | --- | --- | --- |
| Bambury | - | - | * | * | ** | * | * | * | | 7 |
| Han | - | - | * | * | ** | * | - | * | | 6 |
| Auezova | - | - | * | * | - | * | * | * | | 5 |
| S.S.Q. | - | - | * | * | * | * | - | * | | 5 |
| Kaya | - | - | * | * | - | * | * | * | | 5 |
| Lopes | - | - | * | * | - | * | * | * | | 5 |
| Matthew | - | - | * | * | - | * | * | * | | 5 |
| Wang | - | - | * | * | - | * | * | * | | 5 |
| Wiencke | - | - | * | * | - | * | * | * | | 5 |
| Bao | - | - | * | * | * | * | * | * | | 6 |
| Wang | - | - | * | * | * | * | * | * | | 6 |
| Weng | - | - | * | * | - | * | * | * | | 5 |
| Yersal | - | - | * | * | - | * | * | * | | 5 |
| Hao | - | - | * | * | * | * | * | * | | 6 |
| Yang | - | - | * | * | - | * | * | * | | 5 |
| Gan | - | - | * | * | * | * | - | * | | 5 |

* A study could be awarded a maximum of one star for each item except for the item Control for important factor or additional factor. The definition/explanation of each column of the Newcastle-Ottawa Scale is available at <http://www.ohri.ca/programs/clinical_epidemiology/oxford.htm>.
